# Identification of Chlorogenic Acids from *Moringa oleifera* Leaves as Modulators of Prion Aggregation Using Affinity Selection-Mass Spectrometry

**DOI:** 10.1021/acsomega.4c09150

**Published:** 2025-01-15

**Authors:** Magali Silva de Amorim, Manuela Amaral-do-Nascimento, Vanessa Gisele Pasqualotto Severino, Jerson Lima da Silva, Tuane Cristine Ramos Gonçalves Vieira, Marcela Cristina de Moraes

**Affiliations:** † Instituto de Química, Departamento de Química Orgânica, BioCrom, Universidade Federal Fluminense, 24210-141 Niterói, RJ, Brazil; ‡ Instituto de Bioquímica Médica, Instituto Nacional de Ciência e Tecnologia de Biologia Estrutural e Bioimagem, Universidade Federal do Rio de Janeiro, 21941-902 Rio de Janeiro, RJ, Brazil; § Universidade Federal de Goiás, Instituto de Química, 74690-900 Goiânia, GO, Brazil

## Abstract

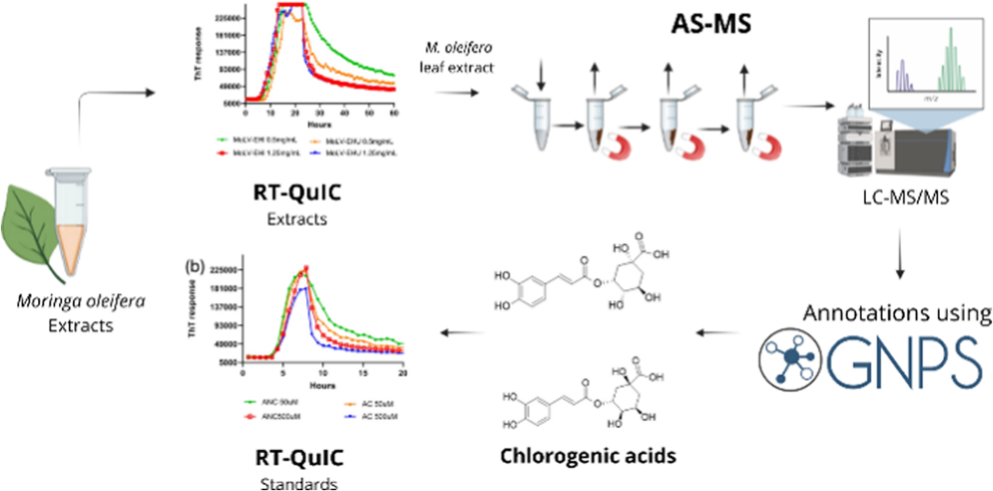

Prion diseases are fatal neurodegenerative disorders caused by the misfolding and aggregation of the cellular prion protein (PrP^C^) into its pathogenic form (PrP^Sc^), leading to progressive neurodegeneration. Currently, no effective treatments are available, highlighting the need for novel therapeutic strategies. In this study, we explored the potential of *Moringa oleifera* extracts as a source of bioactive compounds that could modulate prion protein aggregation. A hydroethanolic extract from *M. oleifera* leaves was analyzed using PrP aggregation inhibition profiling via real-time quaking-induced conversion (RT-QuIC) assays, in combination with affinity selection-mass spectrometry (AS-MS). This approach identified chlorogenic and neochlorogenic acids as potent inhibitors of prion aggregation. These compounds exhibited significant antiprion activity, with IC_50_ values of 64.41 ± 12.12 and 35.34 ± 7.09 μM, respectively. In addition to inhibiting the conversion of PrP^C^ to PrP^Sc^, both compounds could disaggregate preformed PrP^Sc^ fibrils *in vitro*. AS-MS proved to be a valuable tool for isolating the modulators of PrP aggregation directly from crude natural product extracts, avoiding the need for expensive and time-consuming fractionation and purification processes. Identifying chlorogenic and neochlorogenic acids highlights the therapeutic potential of natural products in combating prion diseases and other amyloidogenic disorders. Our findings suggest that these bioactive compounds could serve as promising lead compounds for developing novel treatments for prion diseases. Further *in vivo* studies and pharmacokinetic optimization are warranted to explore their full therapeutic potential.

## 1 Introduction

Amyloidogenic diseases involve the abnormal aggregation of specific proteins into amyloid fibrils, leading to cellular dysfunction and neurodegeneration.^1^ Examples of amyloidogenic proteins include tau in Alzheimer’s, α-synuclein in Parkinson’s, and prion protein in prion diseases.^2^ These latter are particularly unique due to their infectious nature, where misfolded proteins can trigger misfolding of otherwise normal proteins.

Prion diseases are rare, fatal neurodegenerative disorders caused by the conversion of prion protein (PrP^C^) into its pathogenic form (PrP^Sc^), which accumulates in the brain and induces neurodegeneration. Despite their rarity, prion diseases are of profound importance due to their devastating impact on affected individuals, their unique infectious mechanism, and their insight into broader neurodegenerative processes. The search for therapeutic molecules is critical, as no effective treatments currently exist, making prion diseases a significant area of research with the potential to uncover novel strategies for combating these disorders and other protein aggregation diseases.

The factors associated with the initiation and progression of amyloid formation in prion diseases are not fully understood. However, various molecular chaperones, post-translational modifications, and environmental factors are known to influence the process.^3^ Potential therapeutic strategies to combat these disorders involve preventing misfolding, inhibiting aggregation, disrupting existing fibrils, or enhancing the clearance of misfolded proteins.^1^ Emerging approaches, such as antisense oligonucleotides^4^ and epigenetic editing,^5^ focus on decreasing the levels of PrP^C^ to reduce its conversion into PrP^Sc^. While antisense oligonucleotides are currently in clinical trials,^6^ the effectiveness of these strategies may be limited by the extent of protein aggregation in the patient’s brain and the timing of diagnosis, making early intervention crucial. Therefore, small molecules that can modulate the misfolding and aggregation of the PrP^C^ or promote the PrP^Sc^ disaggregation represent promising therapeutic candidates for prion diseases and other amyloidogenic disorders.^7,8^ In this context, natural products have emerged as a rich source of chemical scaffolds with antiaggregation potential, offering novel opportunities for effective therapeutics.^9^ Examples of natural compounds with antiaggregation activity for different amyloidogenic proteins include epigallocatechin-3-gallate, curcumin, resveratrol, and hypericin.^9^

*Moringa oleifera* (Mo), belonging to the Moringaceae family and commonly referred to as the “miracle tree” or “tree of life”, is extensively cultivated worldwide due to its resilience to harsh environmental conditions such as frost and drought. Different parts and formulations of *M. oleifera* have been traditionally used in traditional medicine to treat a wide range of ailments.^10,11^ Recent pharmacological studies have confirmed diverse bioactivities of *M. oleifera* extracts, including anti-inflammatory, antioxidant, anticancer, cardioprotective, antidiabetic, antiviral, antiobesity, antidepressant, hepatoprotective, antimicrobial, and neuroprotective effects.^12−18^ Several studies have focused on its neuroprotective properties, demonstrating potential against neurological disorders such as dementia, stroke, Alzheimer’s, and Parkinson’s diseases.^15,19^ These neuroprotective effects are primarily attributed to its rich phytochemical composition, including flavonoids (e.g., quercetin and kaempferol), phenolic acids (e.g., chlorogenic and caffeic acids), and isothiocyanates, which were previously reported to modulate oxidative stress, neuroinflammation, and apoptosis.^19^

For instance, flavonoids such as quercetin have been reported to inhibit β-amyloid aggregation and reduce tau hyperphosphorylation, mechanisms linked to Alzheimer’s disease.^20^ Similarly, caffeic acid exhibits a broad neuroprotective profile against a diverse range of stressors that result in neuronal cell death.^21^ Kaempferol and its derivatives also contribute to neuroprotection by preventing amyloid fibril deposition (such as Aβ in Alzheimer’s disease and α-synuclein in Parkinson’s disease), inhibiting microglia activation, reducing inflammatory factor release, and restoring mitochondrial membrane function to mitigate oxidative stress.^22^ These findings underscore the therapeutic potential of *M. oleifera* phytochemicals in addressing neurodegenerative diseases through multiple mechanisms.

However, the potential effects of *M. oleifera* on prion protein aggregation have not yet been explored, highlighting a gap in understanding its effectiveness against prion diseases. Addressing this gap requires efficient strategies to identify and characterize bioactive compounds. Traditional screening methods, which rely on repetitive fractionation and biological assays, are often labor-intensive and time-consuming. To address these challenges, innovative assays based on protein–ligand interactions, such as affinity selection-mass spectrometry (AS-MS), have become powerful tools for identifying bioactive compounds directly from crude natural product extracts. The selective interaction between the ligand and the biological target allows for the formation of a target-ligand complex, enabling the isolation and identification of the ligand directly from complex libraries.^23−26^ In AS-MS assays, immobilizing the target onto the surface of magnetic particles facilitates the separation of the protein–ligand complex from unbound compounds by simply using a magnet.^25,27^

The lack of effective therapies for prion diseases underscores a critical gap in treating protein aggregation disorders. This study addresses this gap by investigating bioactive compounds from *M. oleifera*, a plant with known neuroprotective properties, as potential modulators of prion protein aggregation. Using AS-MS and PrP aggregation inhibition profiling, we successfully isolated and identified two bioactive compounds, chlorogenic and neochlorogenic acids, from *M. oleifera* leaf extracts. These compounds demonstrated the ability to inhibit prion aggregation, prevent autopropagation, and disaggregate PrP fibrils in vitro, highlighting their potential as therapeutic agents for prion diseases.

## 2 Results and Discussion

Building on our previous application of the AS-MS approach to identify PrP ligands in a compound mixture using quinacrine as a model,^28^ this study utilizes this promising assay to screen a natural product for prion aggregation modulators. We focused on the neuroprotective potential of *M. oleifera*, known for its rich content of bioactive phytochemicals with neuropharmacological activity.^13^

### 2.1 Screening of *M. oleifera* Extracts Using Real-Time Quaking-Induced Conversion (RT-QuIC)

**Figure 1:**
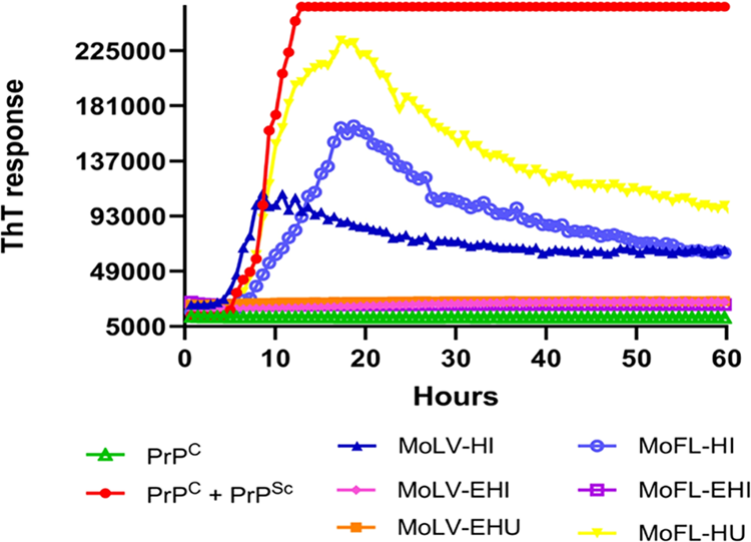
Inhibition of *in vitro* PrP^C^ aggregation with different extracts of *M. oleifera*. Extracts obtained from leaves (MoLV) or flowers (MoFL) at 50 mg/mL were added to PrP^C^ protein in the absence or presence of *in vitro*-produced PrP^Sc^. A sample containing only PrP^C^ and vehicle (MeOH/H_2_O, 1:1) was used as a negative control. A sample containing only PrP^Sc^ and vehicle was used as a positive control for the conversion reaction. The effect of the extracts in the absence of PrP^Sc^ is not shown, as it was similar to the negative control. EHI, hydroethanolic extract prepared by infusion. EHU, hydroethanolic extract prepared by ultrasound. ThT, Thioflavin T.

We initially evaluated the inhibitory activity of six different extracts from the leaves and flowers of *M. oleifera* on prion protein aggregation. These extracts were added to the PrP^C^ substrate before adding PrP^Sc^ fibrils. Negative controls (lacking PrP^Sc^) and positive controls (containing PrP^Sc^ without extracts) were included for comparison. Thioflavin T fluorescence was monitored over 60 h to monitor the progression of fibril formation (Figure 1). We observed that the hydroethanolic extracts from leaves prepared by infusion (MoLV-EHI) and by ultrasound (MoLV-EHU), as well as the hydroethanolic extract from flowers prepared by infusion (MOFL-EHI), completely inhibited the conversion of PrP^C^ to PrP^Sc^. Given the greater availability of leaves compared to flowers and the notable antiaggregation activity exhibited by the MoLV-EHI and MoLV-EHU extracts, these libraries were selected for further investigation.

**Figure 2:**
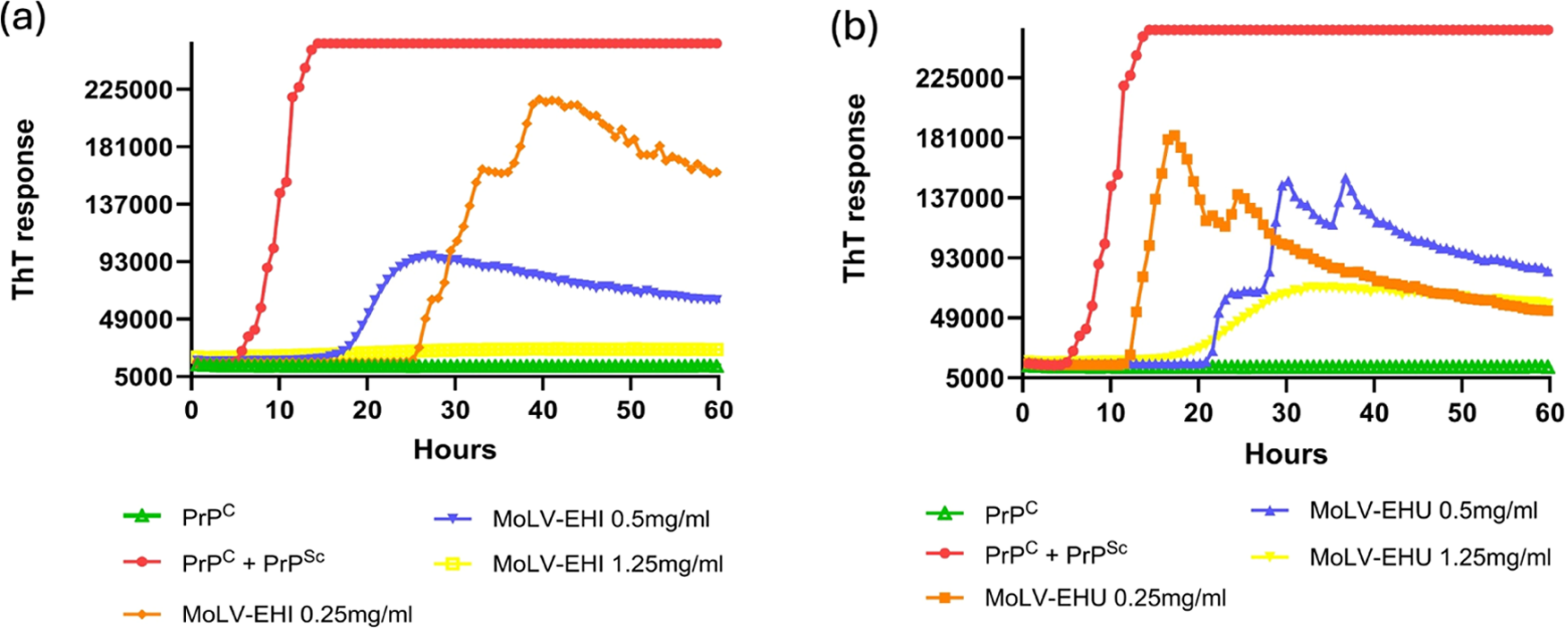
Concentration-dependent inhibition of *in vitro* PrP^C^ aggregation by MoLV-EHI and MoLV-EHU extracts in the RT-QuIC assay. (a) MoLV-EHI extract and (b) MoLV-EHU extract were evaluated at 0.25 mg/mL, 0.5 mg/mL, and 1.25 mg/mL in the presence of *in vitro*-produced PrP^Sc^. PrP^C^ without PrP^Sc^ was used as a negative control, while PrP^C^ with PrP^Sc^ was a positive control for the conversion reaction in the RT-QuIC assay. EHI, hydroethanolic extract prepared by infusion. EHU, hydroethanolic extract prepared by ultrasound. ThT, Thioflavin T.

To confirm that the observed effect was explicitly due to the compound present in the extracts, we evaluated the dose–response patterns of MoLV-EHI and MoLV-EHU extracts. *In vitro* PrP^C^ aggregation inhibition was assessed at three concentrations (0.25, 0.5, and 1.25 mg/mL) for each extract. As shown in Figure 2, both extracts exhibited a concentration-dependent inhibition of PrP^C^ aggregation. Notably, MoLV-EHI completely inhibited PrP^C^ aggregation at 1.25 mg/mL, while MoLV-EHU only partially inhibited aggregation at the same concentration. These results suggest that the infusion extraction technique was more effective in extracting bioactive compounds, leading us to investigate the MoLV-EHI extract further by establishing its inhibition profiling and conducting the AS-MS assay.

### 2.2 Analysis of Prion Aggregation Inhibition and Disaggregase Activity by Extracts from *M. oleifera* Leaves

**Figure 3:**
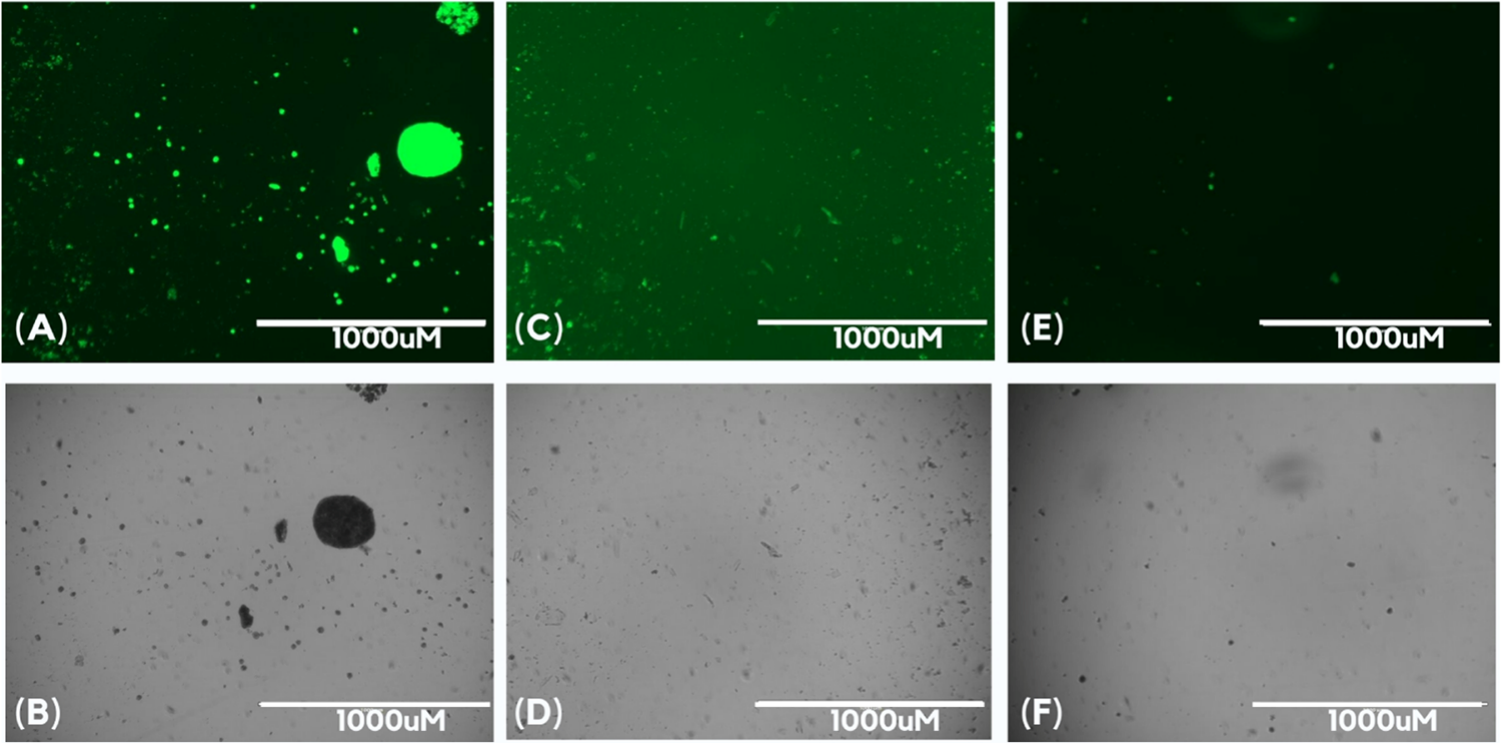
Extracts inhibit protein aggregation without interfering with ThT binding. Representative images from one well per condition were selected from the quadruplicate experiments. (A, B) Positive control, PrP^C^ seeded with PrP^Sc^. (C, D) PrP^C^ with MoLV-EHI extract at 1.25 mg/mL seeded with PrP^Sc^. (E, F) PrP^C^ with MoLV-EHU extract at 1.25 mg/mL seeded with PrP^Sc^. Panels (A, C, E) show fluorescence microscopy images, while panels (B, D, F) display corresponding bright-field microscopy images.

To determine whether the decrease in thioflavin T fluorescence was due to a reduction in PrP^Sc^ formation or interference with the fluorophore binding or fluorescence, we analyzed the RT-QuIC products from the dose–response assay using bright-field and fluorescence microscopy (Figure 3). The extracts’ samples showed a markedly smaller number of ThT-bound aggregates, consistent with the decreased fluorescence observed in the plate reader. Compared to the bright-field images, we also noted a significant reduction in the total number of aggregates, confirming the inhibitory effect of the extracts. If the extracts had interfered with ThT binding, the aggregates would not have been visible under the GFP filter (which detects fluorescence at a wavelength similar to ThT). However, we would still detect it in bright-field microscopy.

**Figure 4:**
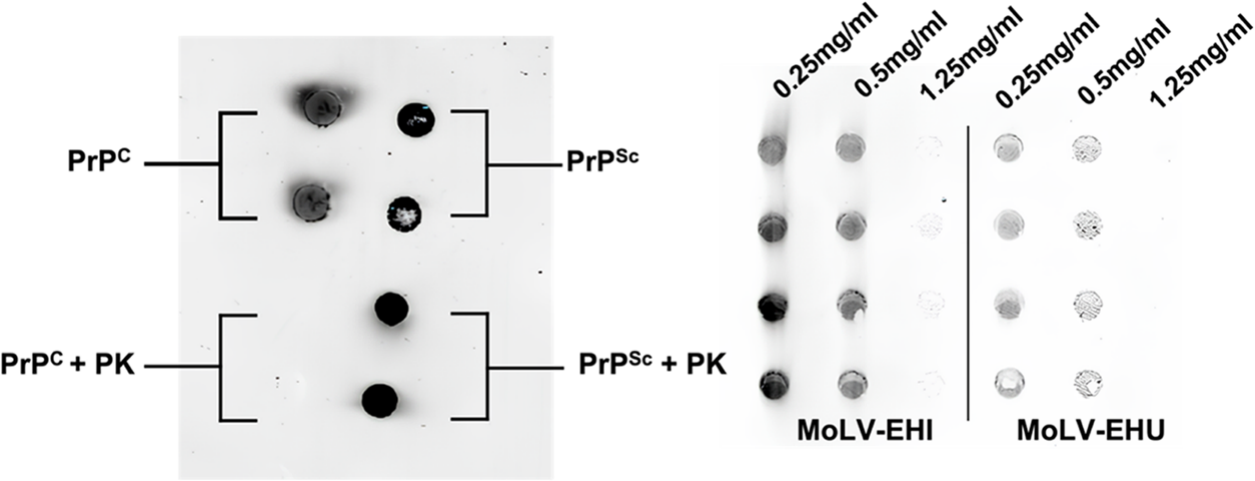
MoLV-EHI and MoLV-EHU extracts reduce PK-resistant PrP^Sc^ levels in a concentration-dependent manner. Dot-blot analysis of PK resistance under various experimental conditions. (Left) Control dot-blot showing the behavior of PrP^C^ and PrP^Sc^ in the presence of proteinase K. After RT-QuIC aggregation cycles, the PrP^C^ sample was sensitive to PK, while the PrP^Sc^ sample remained resistant. (Right) Dot-blot of samples treated with MoLV-EHI and MoLV-EHU at concentrations of 0.25 mg/mL, 0.5 mg/mL, and 1.25 mg/mL, following PK digestion. EHI, hydroethanolic extract prepared by infusion. EHU, hydroethanolic extract prepared by ultrasound. PK, proteinase K.

In addition to visualizing the aggregates via optical microscopy, we conducted a dot-blot analysis on RT-QuIC products following treatment with proteinase K (PK), as PrP^Sc^ is characteristically resistant to this protease (Figure 4). The results revealed increased digestion with rising concentrations, indicating a reduced amount of PK-resistant PrP^Sc^ in these samples, further confirming the antiprion activity of the extracts.

**Figure 5:**
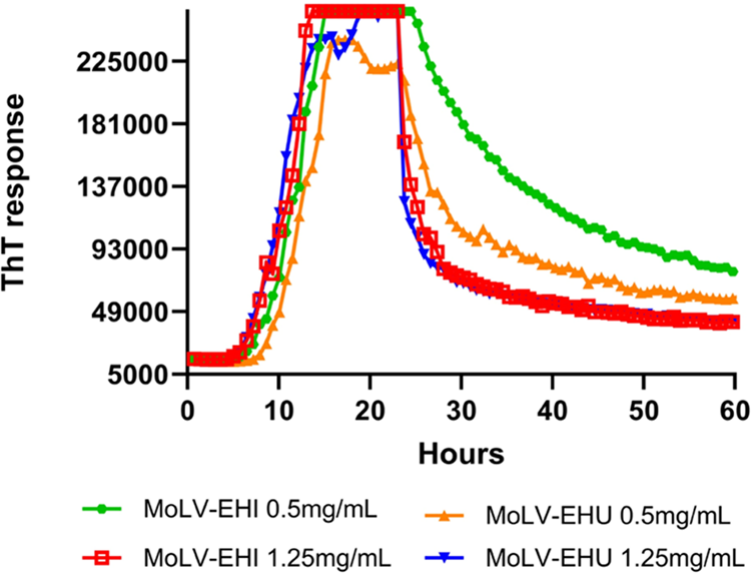
MoLV-EHI and MoLV-EHU promote disaggregation of PrP^Sc^. After 24 h of PrP^Sc^ aggregation in the RT-QuIC assay, MoLV-EHI and MoLV-EHU extracts were added at concentrations of 0.5 and 1.25 mg/mL. ThT fluorescence was monitored for an additional 24 h, showing an exponential decrease over time, indicating disaggregation of PrP^Sc^. EHI refers to the hydroethanolic extract prepared by infusion, while EHU denotes the hydroethanolic extract prepared by ultrasound.

To assess the disaggregation activity of MoLV-EHI and MoLV-EHU extracts, we first performed the RT-QuIC assay without the extracts, allowing aggregates to form until a plateau. After 24 h, MoLV-EHI and MoLV-EHU extracts were added at concentrations of 0.5 and 1.25 mg/mL, and the samples were returned to the plate reader to continue agitation cycles at 55 °C (Figure 5). The results showed a reduction in ThT fluorescence, indicating that both extracts effectively disassembled preformed aggregates.

### 2.3 Inhibition Profiling of the *M. oleifera* Leaf Extract Prepared by Infusion

MoLV-EHI extract exhibited the most promising antiaggregation activity against PrP^C^ in the RT-QuIC assay at 1.25 mg/mL. Consequently, it was selected for further studies to identify the bioactive compounds responsible for this effect. To achieve this, we used two complementary approaches: inhibition profiling and AS-MS assay.

First, we optimized the chromatography conditions to achieve the highest resolution for MoLV-EHI extract analysis, as detailed in the experimental section. Following this, an analytical-scale microfractionation on the MoLV extract was performed using a 60 min gradient elution profile. The extract was injected into the chromatographic system at a concentration of 100 mg/mL, and the eluate was microfractionated every 12 s, resulting in a resolution of 5 points per minute.

**Figure 6:**
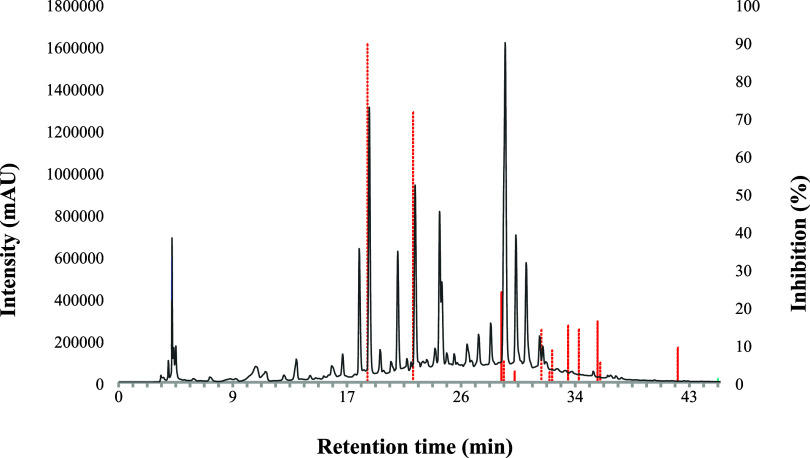
A typical chromatogram for the MoLV-EHI extract (black) superimposed with the PrP aggregation inhibition profile (red). Under optimized chromatographic conditions, 107 peaks were detected at 280 nm. Analytical-scale microfractionation was performed by injecting 20 μL of the MoLV-EHI extract (100 mg/mL) and collecting the eluate into 200 microtubes, yielding one microfraction every 12 s. The red bars represent the percentage of PrP aggregation inhibition, as determined by the RT-QuIC assay for each microfraction. The most potent microfractions, exhibiting over 70% inhibition of PrP aggregation, were collected at 18.5 and 21.9 min.

PrP aggregation inhibition data were plotted as a biochromatogram (Figure 6), illustrating the percentage of inhibition relative to the retention time for each dried microfraction. The data revealed the highest PrP aggregation inhibition (83.3%) at 18.5 min, indicating that the most potent bioactive compound in the MoLV-EHI extract elutes at this retention time. Another significant inhibition (71.3%) was observed at 21.9 min. Other regions between 28 and 43 min showed mild inhibition of PrP aggregation (values ranging from 2.7 to 26%). Therefore, the biochromatogram effectively identified two key microfractions with potent PrP aggregation inhibition (>70%) at 18.5 and 21.9 min in the MoLV-EHI extract.

### 2.4 Affinity Selection-Mass Spectrometry (AS-MS)

AS-MS is a high-throughput screening assay that identifies ligands in complex libraries, such as extracts from natural products. In the first step, the biological target is incubated with the selected library, allowing the ligands in the mixture to interact with the target, forming target-ligand complexes. The second step involves separating these target-ligand complexes from the remaining unbound compounds in the solution. This separation can be facilitated by the prior immobilization of the target onto the surface of magnetic solid supports. Finally, the target-ligand complexes are dissociated, and the ligands are analyzed using mass spectrometry to annotate their chemical structure.

In previous work, we described the immobilization of PrP onto the surface of magnetic particles for ligand recognition in a mixture of quinacrine, caffeine, and thiamine.^28^ This method successfully isolated quinacrine, an inhibitor of PrP aggregation. In this study, we applied a similar AS-MS assay, with minor modifications, to isolate ligands from the MoLV-EHI.

PrP-coated magnetic particles (PrP-MP) were incubated with the MoLV-EHI solution, allowing the formation of PrP-ligand complexes with compounds in the extract that bind to the target biomolecule. These complexes, anchored on the MP surface, were separated from the extract solution using magnets. The MPs were then washed twice with a buffer, and the PrP-ligand complexes were dissociated with methanol. The dissociated ligands were analyzed by LC-HRMS/MS under the same chromatographic conditions established for the study of the PrP aggregation inhibition profile. A control experiment using MPs without immobilized PrP (only coated with glycine) was performed to calculate an affinity ratio for each isolated ligand, excluding nonspecific binding.

Chromatograms obtained from analyzing the supernatants from the AS-MS assay using PrP-MP and the control experiment (Figure S1) evidence the success of this technique in isolating PrP ligands from the MoLV-EHI extract. Several compounds were isolated in higher amounts when the PrP-MPs were employed, instead of only glycine-coated MPs. However, in the AS-MS assay, ligands are isolated based on their affinity for the protein, regardless of their functional effect.^29^ Therefore, the results from the inhibition profile study of this same extract were used to guide the analysis of ligands with the desired functional effect. The biochromatogram in Figure 6 shows that the most active compounds against PrP aggregation elute at 18.5 and 21.9 min. Considering possible variations in retention times due to the different equipment used in these studies, the structures and affinity ratios of ligands with retention times between 17 and 23.5 min were analyzed.

Within this retention time range, two intense peaks were observed at 19.8 and 23.3 min, with an *m*/*z* of 163.0403. Using the tandem mass spectrometry (MS/MS) data and molecular networking (GNPS platform - http://gnps.ucsd.edu), these substances were identified as monocaffeoylquinic acids (chlorogenic and neochlorogenic acids). Table 1 presents the affinity ratio values for the *m*/*z* ratios associated with these ligands.

**Table 1:**
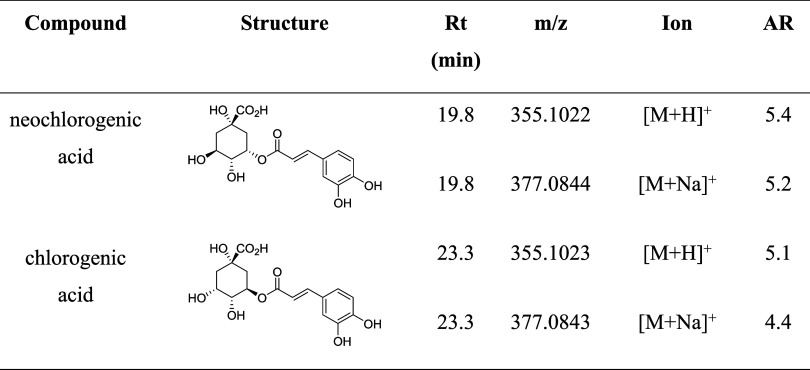
Summary of the AS-MS Assay Results for Screening PrP Ligands in *M. oleifera* Leaf Extract, Focusing on Ligands with Retention times (Rt) between 17 and 23.5 min^a^

a The structure, molecular formula, experimental m/z value, and detected ion for each ligand are listed. AR (affinity ratio) is calculated by dividing the peak area of each EIC from S3 sample in the PrP^C^-MPs assay by the peak area of the corresponding EIC from the S3 sample in the control experiment (glycine-coated MPs).

The base peak associated with these ligands, with an *m*/*z* of 163.0403 and retention times of 19.8 and 23.3 min (Figure S1), corresponds to the ion formed by the dehydration of the caffeic acid portion of both isomers of monocaffeoylquinic acid. The detection of this species can be attributed to the cleavage of the ester bond linking the quinic acid moiety to the caffeic acid moiety, which is susceptible to gas-phase hydrolysis. Additionally, the high temperatures during analysis led to water loss from caffeic acid,^30^ resulting in the ion with an *m*/*z* of 164.0403, as reported in the literature. The ion with an *m*/*z* of 355.102 corresponds to the molecular ion of each isomer, protonated at one of the oxygens, while the ion with an *m*/*z* of 377.084 represents the sodium adduct.

Given the similarity between the mass spectra of these isomers, their unequivocal identification was accomplished by analyzing analytical standards. By injecting pure individual solutions of these compounds, it was determined that neochlorogenic acid (or 3-*O*-caffeoylquinic acid) has a retention time of 19.8 min, while chlorogenic acid (or 5-*O*-caffeoylquinic acid) has a retention time of 23.3 min (Figure S2). Table 1 shows affinity ratio values greater than 4.3 for the detected protonated molecules and sodium adducts related to the two identified ligands. The high-affinity ratio indicates that the ligands were isolated due to specific interactions with the PrP protein, rather than secondary interactions with the solid support used for PrP immobilization.

Chlorogenic and neochlorogenic acids are isomers belonging to the class of naturally occurring compounds known as chlorogenic acids. These compounds are among the most common phenolics in the human diet, found in high concentrations in coffee, tea, fruits, and vegetables. They are associated with various health benefits, including antioxidant, anti-inflammatory, neuroprotective, and central nervous system-stimulating effects.^31^ Studies focusing on the neuroprotective effects of caffeoylquinic acid-related compounds have reported their ability to inhibit the aggregation of 42-residue amyloid β-protein (Aβ42). Structure–activity relationship studies suggest that the caffeoyl group is crucial for this inhibitory activity.^32^ These findings highlight the potential of chlorogenic and neochlorogenic acids as inhibitors of prion protein aggregation.

To gain a deeper understanding of its bioactive potential, the chemical space of MoLV-EHI was assessed using a combination of LC-HRMS/MS and molecular networking, a tool used to identify metabolites based on similarities in their MS-based fragmentation patterns and signals. A total of 18 compounds were annotated, including one nucleoside, one amino acid, five phenolic acids, and 11 flavonoids (Table S1). Since *M. oleifera* is a well-studied plant known for its pharmacological activities, most of these compounds have already been reported in its leaves. However, compound 9, tentatively identified as isoorientin, had only been previously described in the seeds of *M. oleifera*.^33^ Additionally, compound 18, annotated as luteolin 7-(6″-malonylhexose), appears to be reported here for the first time in the leaves of *M. oleifera*, based on our literature review. Although only 2 out of the 18 annotated compounds were identified as PrP ligands, these findings may still contribute to the broader pharmacological effects of *M. oleifera*, such as its antioxidant and neuroprotective properties. This underscores the complex and multifaceted nature of natural extracts, where multiple bioactive compounds may act synergistically to produce therapeutic benefits.

### 2.5 Chlorogenic and Neochlorogenic Acids Are Inhibitors of PrP Aggregation

**Figure 7:**
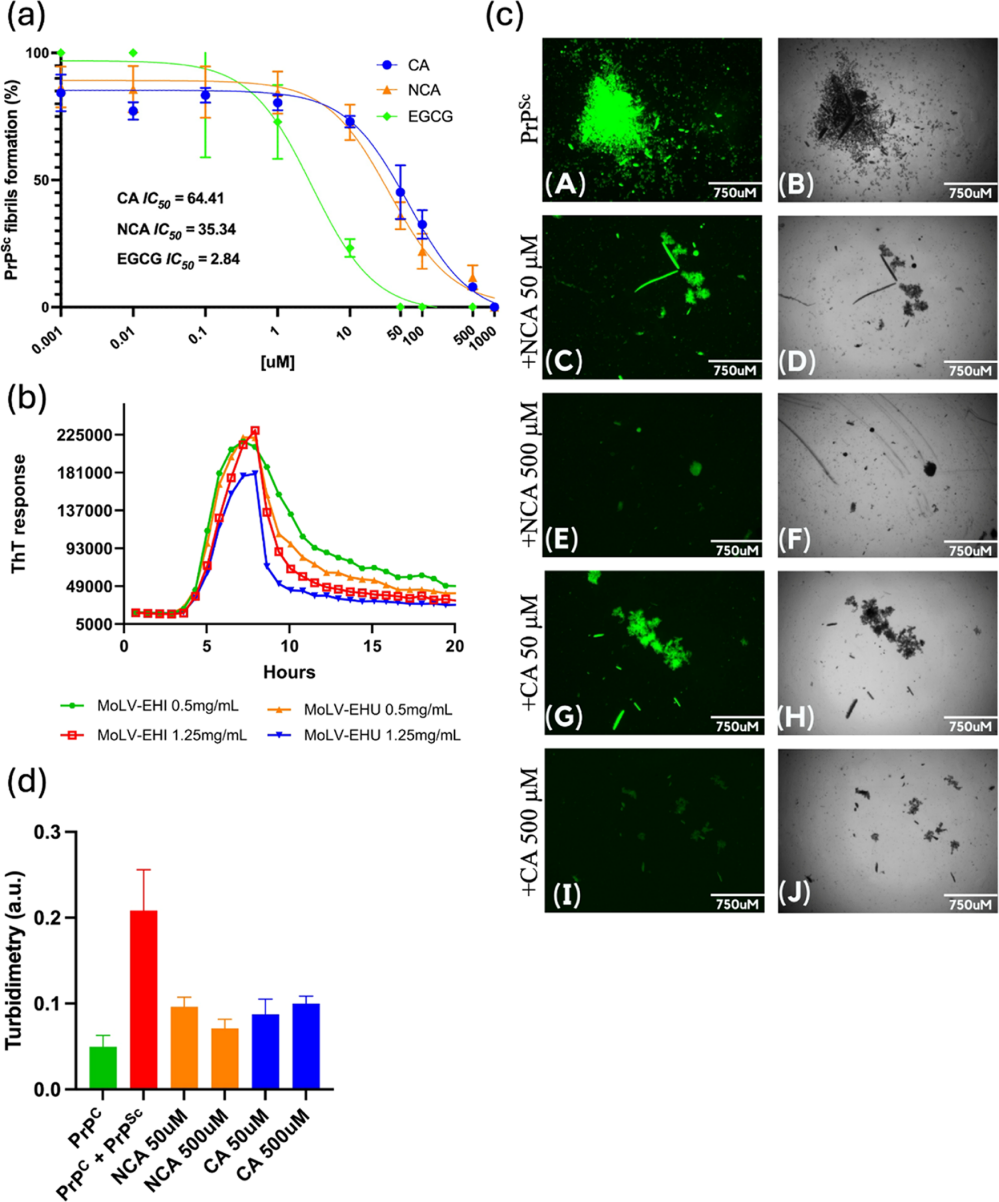
Inhibition of PrP^Sc^ fibrils formation and disaggregation by chlorogenic acid (CA) and neochlorogenic acid (NCA). (a) The graph shows the dose-dependent inhibition of PrP^Sc^ fibril formation by chlorogenic acid (CA, blue circles), neochlorogenic acid (NCA, orange triangles), and epigallocatechin gallate (EGCG, green circles). The percentage of PrP^Sc^ fibrils formed was measured across a range of compound concentrations (0.001 to 1000 μM). The IC_50_ values are 64.41 ± 12.12 μM for CA, 35.34 ± 7.09 μM for NCA, and 2.84 ± 0.97 μM for EGCG. Data are presented as mean ± standard error from three independent experiments in quadruplicate. (b) After 8 h of PrP^Sc^ aggregation in the RT-QuIC assay, CA and NCA were added at 50 μM and 500 μM. ThT fluorescence was monitored for an additional 12 h, showing an exponential decrease over time, indicating disaggregation of PrP^Sc^. (c) (A, B) Positive control, PrP^C^ seeded with PrP^Sc^. (C, D) Addition of 50 μM and (E, F) 500 μM NCA seeded with PrP^Sc^. (G, H) Addition of 50 μM and (I, J) 500 μM CA seeded with PrP^Sc^. Panels (A, C, E, G, I) show fluorescence microscopy images, while panels (B, D, F, H, J) display corresponding bright-field microscopy images. (d) Turbidity at 600 nm of the samples at the end of the RT-QuIC.

To assess the inhibitory effects of chlorogenic (CA) and neochlorogenic acids (NCA) on PrP^Sc^ aggregation, we conducted a dose–response RT-QuIC assay (Figure 7A). The results demonstrated that the chlorogenic acids effectively inhibited *in vitro* PrP^Sc^ conversion and autopropagation within the midmicromolar range, with calculated IC_50_ values of 64.41 μM for CA and 35.34 μM for NCA. This inhibitory effect was notably more robust than that observed with chalcones, heparin, and oxadiazoles in similar RT-QuIC studies.^34−36^ The calculated IC_50_ values for CA and NCA are within the range reported for rosmarinic acid, a polyphenol found in rosemary, in the ScN2A cell culture assay.^37^

We also evaluated the disaggregation potential of CA and NCA (Figure 7B). Similar to the effects of *M. oleifera* extracts, both compounds could reverse PrP^Sc^ aggregation by disassembling preformed fibrils and producing smaller aggregates under the RT-QuIC conditions of agitation and heating. This disaggregation was confirmed through microscopy (Figure 7C) and turbidimetry measurements (Figure 7D). Microscopy images revealed that the size of the aggregates varied depending on the concentration of the compounds. This disaggregation activity is comparable to the well-documented effects of epigallocatechin gallate (EGCG), known for its ability to break down amyloid fibrils into nontoxic species.^38^ Given the difficulty of reversing protein aggregation, this promising activity could mitigate or reverse the damaging effects of prion and other amyloidogenic diseases.

For comparison, a similar dose–response RT-QuIC assay using EGCG, a well-characterized polyphenol from green tea, yielded an IC_50_ value of 2.84 μM under the same conditions, demonstrating a significantly stronger inhibitory effect on PrP^Sc^ aggregation. While EGCG exhibits a lower IC_50_, CA and NCA offer unique advantages, including better bioavailability and multifunctional therapeutic potential.^31,39^ In addition, EGCG also effectively inhibited the accumulation of cell PrP^Sc^ at 50 μM,^40^ indicating the potential for testing CA and NCA *in cellular* and *in vivo* models.

As well as chlorogenic acids, EGCG also has anti-inflammatory and antioxidant properties, and neuroprotective effects. EGCG acts through a multitarget mode of action, synergistically addressing protein misfolding, oxidative stress, apoptosis, and neuroinflammation. Although studies have demonstrated that EGCG interacts with various amyloidogenic proteins, including Aβ, tau, α-synuclein, transthyretin, and huntingtin,^38,41,42^ clinical evidence supporting the antineurodegenerative effects of EGCG remains limited. No impact on disease progression was observed in a clinical phase study involving patients with multiple system atrophy—characterized by the aggregation of α-synuclein in oligodendrocytes and neurons. Additionally, some patients experienced hepatotoxic effects, leading to treatment discontinuation.^43^ This inconsistency in results may be attributed to the poor pharmacokinetic properties and bioavailability of catechins.^44^

However, chlorogenic acids offer several potential advantages over EGCG. Studies have shown that chlorogenic acids, like those identified in this study as PrP aggregation inhibitors, are more readily absorbed and metabolized in humans.^39^ For example, the bioavailability of chlorogenic acid has been enhanced through intranasal administration, significantly increasing its brain concentration in animal models.^45^ This delivery method could potentially overcome one of the significant challenges polyphenolic compounds like EGCG face, which often fail to reach therapeutic concentrations in the central nervous system.

Furthermore, CA and NCA possess potent antioxidant and anti-inflammatory properties, crucial in neurodegenerative conditions characterized by oxidative stress and chronic inflammation.^31^ Their dual role in modulating protein aggregation and reducing oxidative damage makes them particularly attractive as multitarget therapeutics. This multifunctionality aligns with emerging therapeutic strategies that seek to address multiple pathological processes simultaneously rather than targeting a single disease mechanism.

Given the promising *in vitro* results for both CA and NCA, further studies are warranted to evaluate their efficacy *in vivo*. Future research should focus on optimizing their pharmacokinetic profiles and determining the most effective delivery methods for targeting the central nervous system. Additionally, exploring these compounds’ structure–activity relationship (SAR) could help identify more potent derivatives or analogs with enhanced antiaggregation activity and better bioavailability.

In terms of broader implications, the ability of these compounds to inhibit prion protein aggregation suggests that they could be repurposed for other protein-misfolding diseases, such as Alzheimer’s, Parkinson’s, and Huntington’s. The commonality in protein aggregation pathways across these disorders provides a rationale for investigating CA and NCA as potential therapeutic agents in neurodegenerative diseases. Moreover, identifying these compounds from natural products reinforces the value of traditional medicinal plants as sources of novel bioactive molecules with therapeutic potential.

## 3 Conclusions

This study highlights the remarkable potential of AS-MS combined with inhibition profiling in identifying bioactive compounds directly from complex crude extracts, bypassing traditional fractionation and purification methods. Using this screening platform, we successfully identified chlorogenic and neochlorogenic acids as key modulators of PrP aggregation from *M. oleifera* leaf extract. These compounds effectively inhibited *in vitro* PrP^Sc^ formation, with IC_50_ values of 64.41 ± 12.12 μM for chlorogenic acid and 35.34 ± 7.09 μM for neochlorogenic acid. Additionally, both compounds demonstrated significant disaggregation of preformed PrP^Sc^ fibrils, as confirmed by microscopy and turbidity measurements, further supporting their potential as antiprion agents. This work represents the first application of AS-MS to identify modulators of protein aggregation in natural products, providing a valuable tool for accelerating the development of new therapies for amyloidogenic diseases.

Additionally, given that protein misfolding and aggregation are common features in several neurodegenerative disorders, including Alzheimer’s and Parkinson’s diseases, chlorogenic and neochlorogenic acids may also hold promise as broader therapeutic agents for amyloid-related conditions. Future studies should focus on evaluating the *in vivo* efficacy of these compounds, optimizing their pharmacokinetic properties, and exploring potential delivery methods to enhance their bioavailability, particularly in the central nervous system. A deeper investigation into these compounds’ structure–activity relationship could also identify more potent analogs or derivatives with enhanced therapeutic potential.

## 4 Materials and Methods

### 4.1 Reagents and Chemicals

All chemicals used during the enzyme immobilization and AS-MS procedure were reagent or analytical-grade. Glutaraldehyde (grade II, 25% in H_2_O (v/v)), pyridine (≥99%), sodium chloride, formic acid LC-MS grade, amino-terminated magnetic particles (50 mg/mL), epigallocatechin gallate, chlorogenic, and neochlorogenic acid were purchased from Sigma (São Paulo, Brazil). Methanol (HPLC grade) was from J.T. Baker (Xalostoc, Mexico). Ultrapure water was obtained in a Milli-Q Direct 8 system (Millipore, São Paulo, Brazil).

### 4.2 Plant Collection and Extract Preparation

Leaves and flowers of *Moringa oleifera* (Mo) were collected in Catalão, Goias, Brazil. A voucher specimen was deposited at the Herbarium of the Federal University of Goiás (UFG) under the number 68186. The genetic resource access was registered under SisGen (AB29947).

The plant material was dried at room temperature and macerated. Extracts from different parts of the plant were prepared either by infusion or ultrasound-assisted extraction. For the infusion technique, an ethanol/water solution (7:3, v/v) or ultrapure water was heated in a bath at 90 °C (SW23 - Julabo) for 1 h. For the ultrasound extraction, only an ethanol/water solution (7:3, v/v) was used as the solvent, and the extract was prepared using ultrasound (Eco-Sonics Q3.8/40) for 20 min at room temperature. The mixtures obtained from both techniques, using either the leaves or flowers of *M. oleifera*, were allowed to cool for 10 min and then filtered. The organic solvent was removed using a rotary evaporator, and the remaining aqueous extract was frozen and lyophilized. Before chromatographic analysis, all extracts were filtered through a Valuprep PTFE syringe filter (0.22 μm).

### 4.3 Prion Protein Expression and Purification

The murine recombinant full-length prion protein (PrP23-231) and hamster truncated prion protein (PrP90-231) were expressed in *Escherichia coli* and purified by high-affinity chromatography according to a previously described protocol.^46^

### 4.4 Screening of *M. oleifera* Extracts Using Real-Time Quaking-Induced Conversion (RT-QuIC)

In this experiment, recombinant PrP90-231 (0.15 mg/mL) was added to a reaction buffer (PBS, 300 mM NaCl, 1 mM EDTA, 10 μM thioflavin T), with or without 4% of *in vitro* produced PrP^Sc^, which self-propagates in the solution over time. These PrP^Sc^ seeds were made after one round of RT-QuIC (24h) using 20 μL of cerebrospinal fluid from a patient diagnosed with CJD. This mixture was added to a 96-well plate and incubated with alternating cycles of agitation (700 rpm) and rest at 55 °C in a fluorescence plate reader FLUOstar OMEGA (BMG Labtech). Thioflavin T is a β-sheet intercalator that fluoresces upon binding to amyloid fibers. An increased fluorescence signal indicates amyloid formation. Analyses were conducted in quadruplicates for increased reliability, and the average of these quadruplicates was graphically represented.

### 4.5 Dose–Response Assays

The extracts from *M. oleifera* leaves obtained using ultrasound (MoLV-EHU) and infusion (MoLV-EHI) techniques exhibited the most promising results in the initial screening. Consequently, dose–response curves were obtained for these extracts by assessing PrP aggregation using the RT-QuIC assay at different concentrations of MoLV extracts (0.25, 0.5, and 1.25 mg/mL), in quadruplicate. The ThT signal from seeded PrP samples in the absence of the MoLV extract was set as the 100% control. ThT responses of seeded samples in varying MoLV extract concentrations were plotted relative to this control. Each condition was tested in three independent experiments, each performed in quadruplicate.

To determine the IC_50_ values for epigallocatechin gallate, chlorogenic acid, and neochlorogenic acid on PrP^Sc^ fibril formation, a similar dose–response assay was conducted. Solutions of epigallocatechin gallate and chlorogenic acids were prepared separately in mixture of MeOH:H_2_O (1:1, v/v), over a concentration range of 0.001 to 1000 μM. Control assays were performed by replacing the inhibitor solution with MeOH:H_2_O (1:1, v/v). The IC_50_ values were determined using nonlinear regression analysis, [Inhibitor] vs. response (three parameters) least-square fit, in Prism software.

### 4.6 Bright-Field and Fluorescence Microscopic Analysis

After the RT-QuIC assay using different concentrations of MoLV-EHI and MoLV-EHU extracts, the samples containing the extract at 1.25 mg/mL were analyzed by bright-field and fluorescence microscopy using EVOS image system (Thermo Scientific) to visualize aggregates. Bright-field and fluorescence microscopy was used to analyze chlorogenic acids disaggregation effect at 50 and 500 μM.

### 4.7 Dot-blot

RT-QuIC products from the dose–response experiment with extracts were digested with proteinase K (10 μg/mL) for 90 min at 37 °C. The reaction was terminated by adding Pefabloc at 0.1 μM. After digestion, the content of each quadruplicate was diluted four times with TBS (20 mM Tris-HCl and 150 mM NaCl, pH 7.5) and transferred to the dot-blot apparatus. The membrane was incubated with 3 M guanidine for 8 min, washed four times with TBS for 15 min, and blocked with Intercept (TBS) Blocking Buffer for 1 h at room temperature. Subsequently, the membrane was incubated with the 6D11 antibody (1:7500) for 1 h and washed three times with TBS-T (TBS solution containing 0.05% Tween 20) for 15 min. Finally, the membrane was incubated with the secondary antibody (1:10,000) (IRDye 800CW Goat anti-Mouse IgG Secondary Antibody) in the dark for 1 h and washed again with TBS-T, four times for 15 min. The membrane was left to dry in the dark, and the image was acquired the following day.

### 4.8 Disaggregase Activity Assays

The RT-QuIC assays previously described were initiated in the absence of the extracts until the ThT response reached its maximum and equilibrium (around 24 h). After this period, both MoLV- extracts were added at two different concentrations (0.5 and 1.25 mg/mL), and the ThT response was continuously monitored. A similar experiment was performed with chlorogenic and neochlorogenic acids at 50 and 500 μM. Once the aggregation reached its maximum (around 8 h), the compounds were added to each well, and the ThT response was continuously monitored. Then, the samples were analyzed by bright-field and fluorescence microscopy using the EVOS image system (Thermo Scientific), and turbidity was measured using CLARIOstar plus plate reader (BMG Labtec), monitoring at 600 nm.

### 4.9 Inhibition Profiling of the Most Active Extract

Microfractionation of the most active extract (MoLV-EHI) was conducted using a Shimadzu LC 20 AD XR liquid chromatography system (Kyoto, Japan), which included an LC 20 AD XR pump, an autosampler equipped with a 100 μL Loop (SIL 20 A XR), a column oven (CTO-20A), and a variable wavelength detector connected via a SHIMADZU SCL 20 AD XR interface. Chromatograms were recorded using LC Solutions Software (LabSolutions 5.84 software). The chromatographic conditions were established based on a previously published study on the chromatographic analysis of hydroethanolic extracts from *M. oleifera* flowers.^47^ A Supelco Ascentis C18 column (25 cm × 4.6 mm, 5 μm) was used with a gradient elution employing 0.1% formic acid in water (solvent A) and methanol (solvent B), at a flow rate of 0.8 mL/min. The total run time was 60 min, with the following gradient profile: 0–5 min, 3% B (isocratic); 5–40 min, 3–97% B (linear gradient); 40–45 min, 97% B (isocratic); 45–50 min, 97–3% B (linear gradient); 50–60 min, 3% B (isocratic). A 20 μL sample of the MoLV-EHI extract (100 mg/mL in MeOH/H_2_O, 1:1, v/v) was injected. Once the conditions were established, the eluate was collected in 2 mL microtubes at intervals of 12 s. The eluates were dried using a Speed-Vac concentrator from the Genevac miVac model.

We assessed the prion antiaggregation activity of each dried microfraction using the previously described RT-QuIC assay. Each microfraction was resuspended in 20 μL of MeOH:H_2_O (1:1, v/v), and 5 μL was added to each well before PrP^Sc^. The ThT signal from seeded PrP samples in the absence of the microfraction sample was set as 100%. The ThT responses of seeded samples in the presence of microfraction samples were then plotted relative to this control. The inhibition profile was displayed as a biochromatogram, plotting the percentage of PrP^Sc^ aggregation inhibition against the retention time of each microfraction.

### 4.10 Protein Immobilization

PrP was covalently immobilized onto the surface of amine-terminated magnetic particles (MP, 1 μm, superparamagnetic iron oxide magnetic particles, Sigma-Aldrich, CAS 105808-72-8) according to a previously published protocol.^28^ Briefly, 60 μL of the MP suspension at 50 mg/mL was washed three times with 1 mL of coupling buffer (0.01 M pyridine, pH 6.0). Next, 1.0 mL of glutaraldehyde (5% in coupling buffer) was added to the mixture and incubated for 3 h on an orbital shaker at room temperature. The MP suspension was washed five times with 1 mL of coupling buffer. Subsequently, 1 mL of PrP^C^ solution (1.0 mg/mL, in coupling buffer) was incubated with the activated MPs overnight at 4 °C on a revolver-type orbital shaker. After that, 1 mL of glycine (1.0 M, pH 8,0) was added to react for 30 min at 4 °C to quench all residual aldehyde groups. The PrPC-coated MP suspension was then stored in a refrigerator until needed for AS-MS assays.

MPs-blank were prepared using a similar procedure but without the PrP^C^ immobilization step. Therefore, the MPs were washed three times with coupling buffer, activated with glutaraldehyde, washed five times with coupling buffer, and finally reacted with glycine.

### 4.11 Affinity Selection-Mass Spectrometry (AS-MS)

AS-MS assays were conducted with MoLV-EHI extract at 4 mg/mL, prepared in MeOH:H_2_O (1:1, v/v). The conditions employed in this study were based on protocols previously established with minor modifications.^28^ Briefly, 5 mg of PrP^C^-MPs were incubated with 1 mL of the MoLV-EHI extract for 1 min using a vortex, followed by 9 min of agitation on a revolver-type orbital shaker at 4 °C. After incubation, the supernatant (S0) was removed. The PrP^C^-MPs were washed twice with 1 mL 5 mM ammonium acetate buffer solution (pH 7.4) and vortexed for 1 min each time. The supernatants from these washes were removed and labeled S1 and S2, respectively. In the final step, PrP^C^-MPs were washed with 1 mL of MeOH for ligand elution, and the microtube was shaken for 3 min on a vortex. The supernatant from this step was collected and labeled S3. Control experiments were conducted using MPs-blank. All supernatant separations were performed by magnetic separation using a neodymium magnet.

The supernatants collected from both assays were analyzed using a UHPLC-HRMS chromatographic system. The selectivity of the isolated ligands (affinity ratio) was determined by dividing the peak area of each Extracted Ion Chromatogram (EIC) from the S3 fraction of the PrP^C^-MPs assay by the peak area of the corresponding EIC from the S3 fraction of the control experiment.

### 4.12 Data-Dependent LC-ESI-HRMS/MS Analysis

The analyses were performed at the Center for Mass Spectrometry of Biomolecules (CEMBIO) at the Federal University of Rio de Janeiro (UFRJ) using the same chromatographic conditions described in the microfractionation study. A liquid chromatography system coupled to a high-resolution mass spectrometer (UHPLC-HRMS), Maxis Impact from Bruker Daltonics, was used to analyze the supernatants collected in the AS-MS assay. A postcolumn split maintained a flow rate of 0.3 mL min^–1^ into the mass spectrometer source. The LC-MS system includes a QqTOF (quadrupole time-of-flight) analyzer operating in DDA/AutoMS acquisition mode, with isolation/fragmentation of 5 precursors/cycle, in a scan range of 50–1200 *m*/*z*, 1 Hz acquisition rate for Full Scan and 2 Hz for DDA, with positive ESI mode, nebulizer set at 4 bar, dry gas at 8 L/min, and dry temperature at 200 °C. The capillary voltage was 3500 V, and the end plate offset at −500 V with quadrupole low mass set at 80 *m*/*z*. The collision cell energy operated at 6 eV, with a transfer time of 50 and 3 μs prepulse storage time. Data were analyzed using Compass DataAnalysis (Bruker, Germany).

The data obtained in the UHPLC-HRMS were processed using MZmine 3 v3.9 software.^48^ Molecular networks were created using the online workflow of the Global Natural Products Social molecular networking platform^49^ (http://gnps.ucsd.edu).

## Supporting Information Available

Base peak chromatograms of the isolated ligands from *M. oleifera* leaf extract; HPLC-DAD chromatograms of chlorogenic acid and neochlorogenic acid standards; table of annotated compounds from MoLV-EHI extract with the corresponding discussion (PDF)
