# Supplementary material for: Identification of Chlorogenic Acids from Moringa oleifera Leaves as Modulators of Prion Aggregation Using Affinity Selection-Mass Spectrometry
Source: ACS Omega. 2025 Jan 15;10(3):2919–30. doi: 10.1021/acsomega.4c09150 (PMC11780439; doi:10.1021/acsomega.4c09150)
Supplement: Supplementary file 1 — ao4c09150_si_001.pdf [file ao4c09150_si_001.pdf]

Identification of chlorogenic acids from *Moringa oleifera* leaves as modulators of prion aggregation using affinity selection-mass spectrometry

Magali Silva de Amorim[a#], Manuela Amaral-do-Nascimento[b#], Vanessa Gisele Pasqualotto Severino[c], Jerson Lima da Silva[b], Tuane Cristine Ramos Gonçalves Vieira[b*], Marcela Cristina de Moraes[a*]

[a]*Universidade Federal Fluminense, Instituto de Química, Departamento de Química Orgânica, BioCrom, 24210-141 Niterói, RJ, Brazil*

[b]*Universidade Federal do Rio de Janeiro, Instituto de Bioquímica Médica, Instituto Nacional de Ciência e Tecnologia de Biologia Estrutural e Bioimagem, 21941-902 Rio de Janeiro, RJ, Brazil*

[c]*Universidade Federal de Goiás, Instituto de Química, 74690-900 Goiânia, GO, Brazil*

SUPPORTING INFORMATION

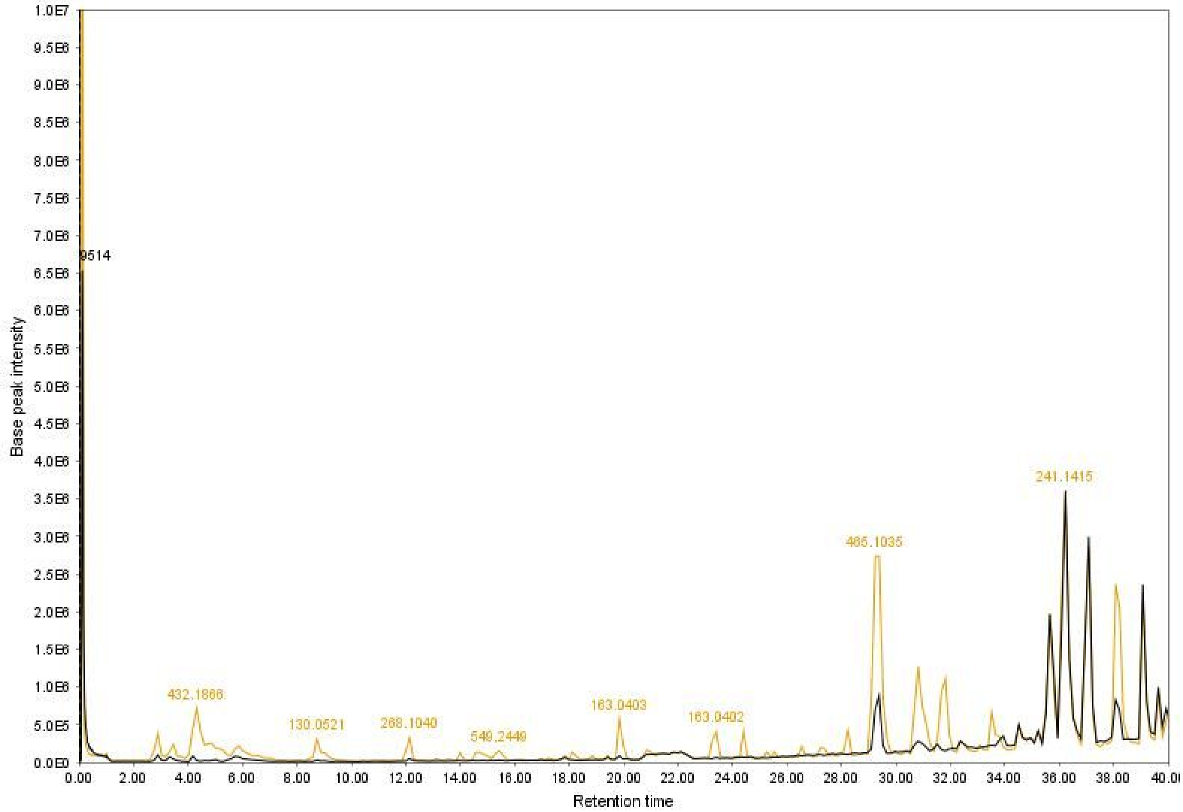

Figure S1. Base peak chromatogram of compounds isolated from *M. oleifera* leaf extract using PrP-coated magnetic particles (yellow) compared to the control assay (black). The most intense peaks in yellow represent ligands selectively isolated due to their affinity for the PrP protein.

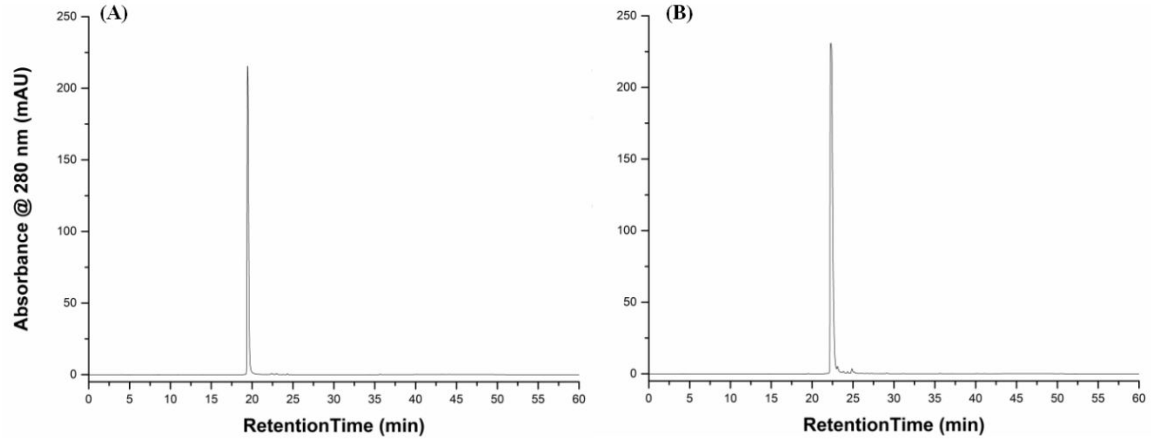

Figure S2. HPLC-DAD chromatograms of neochlorogenic acid (A) and chlorogenic acid (B) standards at 500 µM analyzed under the same conditions used for the sample extracts. The retention times for neochlorogenic acid (3-*O*-caffeoylquinic acid) and chlorogenic acid (5-*O*-caffeoylquinic acid) were 19.8 minutes and 23.3 minutes, respectively. This confirmed the elution order and enabled the structural identification of the isolated ligands from the MoLV-EHI extract using AS-MS assay.

Table S1. Annotated compounds from the hydroethanolic extract of *M. oleifera* leaves. LC-HRMS/MS data acquired in both positive and negative ionization modes were considered at MS^1 and MS^2 levels.

Entry	RT (min)	Compound	Molecular formula	Parent ion *m/z (MS^1)*	Error (ppm)	Fragment ions (*m/z*)
1	12.1	adenosine	$C_{10}H_{13}N_5O_4$	268.1052 $[M+H]^+$	3.7	136.0641
2	17.2	tryptophan	$C_{11}H_{12}N_2O_2$	205.0977 $[M+H]^+$	2.6	188.0716; 146.0619
3	19.8	neochlorogenic acid	$C_{16}H_{18}O_9$	355.1022 $[M+H]^+$	-0.4	163.0403; 89.0424
4	22.1	3-*p*-coumaroylquinic acid	$C_{16}H_{18}O_8$	337.0932 $[M-H]^-$	0.9	163.0405; 119.0503; 191.0567
5	22.7	3-feruloylquinic acid	$C_{17}H_{20}O_9$	367.1030 $[M-H]^-$	-0.4	193.0508
6	23.3	chlorogenic acid	$C_{16}H_{18}O_9$	355.1023 $[M+H]^+$	-0.2	163.0409; 135.0464
7	24.4	vicenin 2	$C_{27}H_{30}O_{15}$	593.1544 $[M-H]^-$	0.1	353.0674; 473.1094; 383.0779
8	25.5	4-*p*-coumaroylquinic acid	$C_{16}H_{18}O_8$	337.0934 $[M-H]^-$	1.5	173.0461; 163.0403; 155.0353
9	26.5	isoorientin	$C_{21}H_{20}O_{11}$	447.0933 $[M-H]^-$	0	327.0517; 357.0564; 297.0388
10	26.7	quercetin-3,4-*O*-di-β-hexose	$C_{27}H_{30}O_{17}$	625.1415 $[M-H]^-$	0.7	463.0880; 301.0354
11	27.2	vitexin	$C_{21}H_{20}O_{10}$	431.0980 $[M-H]^-$	-0.8	311.0563;

						283.0616; 341.0659
12	28.2	isovitexin	$C_{21}H_{20}O_{10}$	431.0992 $[M-H]^-$	1.9	311.0567; 341.0675; 283.0612; 353.0670
13	29.1	rutin	$C_{27}H_{30}O_{16}$	611.1622 $[M+H]^+$	2.5	303.0515; 465.1042; 129.0571
14	30.4	quercetin-3-*O*-hexose-6''-acetate	$C_{23}H_{22}O_{13}$	507.1167 $[M+H]^+$	6.0	303.0506
15	30.8	kaempferol-7-*O*-hexose	$C_{27}H_{30}O_{15}$	595.1672 $[M+H]^+$	2.4	287.0557; 449.1081
16	30.95	isorhamnetin-3-*O*-hexose	$C_{28}H_{32}O_{16}$	625.1801 $[M+H]^+$	6.0	317.0665; 479.1197
17	31.7	quercetin 3-O-malonylhexose	$C_{24}H_{22}O_{15}$	549.0897 $[M-H]^-$	2.0	505.1003; 300.0287
18	33.5	luteolin 7-*O*-(6''-malonylhexose)	$C_{24}H_{22}O_{14}$	533.0941 $[M-H]^-$	0.8	489.1044; 285.0400

Table S1 lists the compounds identified from UHPLC-ESI-QTOF-MS data of *M. oleifera* leaf extract, prepared using ultrasound-assisted extraction with ethanol:water (70:30, v/v). The data were processed using the MZmine 3 v3.9 software,[1] and the structures were subsequently annotated through the online platform Global Natural Product Social Molecular Networking[2] (http://gnps.ucsd.edu). Only peaks that met the following criteria were considered: a cosine score of 0.90 or higher, indicating high similarity between the experimentally obtained mass spectrum and the GNPS spectral library, minimizing the risk of false positives; six or more shared peaks; and a mass difference of 5 ppm or less.

18 compounds were identified based on both positive and negative ionization modes. Compound **1** was tentatively identified as adenosine, eluting at 12.1 minutes. The mass spectrum of this purine nucleoside showed signals at m/z 268.1052 and 136.0641, corresponding to the $[M+H]^+$ precursor and the protonated adenine after the loss of the ribose group, respectively.[3]

Compound **2**, tentatively characterized as the L-tryptophan (Trp), eluted at 17.2 minutes. The mass spectrum of this amino acid provided a signal at m/z 205.0977, corresponding to protonated Trp, which dissociated to form a fragment ion at m/z 188.0716 after losing ammonia. A second abundant fragment at m/z 146.0619 resulted from further loss of CH_2CO.[4]

Compound **3** was identified as neochlorogenic acid (3-*O*-caffeoyl quinic acid), a member of the chlorogenic acid family. It eluted at 19.8 minutes and showed $[M+H]^+$ ions at m/z 355.1022. The base peak at m/z 163.0403 corresponds to the ion formed by the dehydration of the caffeic acid moiety. The second most abundant fragment at m/z 89.0424 was formed after further loss of 2H from the caffeic acid moiety.[5] The structural elucidation of this compound was conducted by analyzing the analytical standard, since this compound was identified as a modulator of PrP aggregation in this study.

Two compounds were identified as *p*-coumaroylquinic acid isomers: compound **4** (3-*p*-coumaroylquinic acid) eluting at 22.1 minutes and **8** (4-*p*-coumaroylquinic acid) eluting at 25.5 minutes, both showing $[M-H]^-$ precursor ions at m/z 337.0932 and 337.0934, respectively. In the negative ionization mode, the peak base of the mass spectrum can be used to differentiate these chlorogenic acids.[6] Compound **4** had a base peak at m/z 163.0405 (deprotonated *p*-coumaric acid), while compound **8** showed a base peak at m/z 173.0461 (deprotonated quinic acid).

Compound **5** was identified as 3-feruloylquinic acid, a chlorogenic acid, with a retention time of 22.7 minutes, showing $[M-H]^-$ ions at m/z 367.1030. The base peak at m/z 193.0508 corresponds to the ferulic acid fragment, suggesting its tentative identification.[6]

Compound **6,** identified as chlorogenic acid (5-*O*-caffeoyl quinic acid), eluted at 23.3 minutes. It showed $[M+H]^+$ ions at m/z 355.1023, with the base peak observed in the mass spectrum at m/z 163.0409 corresponding to the dehydration of the caffeic acid moiety. The fragment ion with a peak at m/z 135.0464 was attributed to $[\text{caffeic acid} - H - CO_2]^-$.[7] The structural elucidation of this compound was conducted by analyzing the analytical standard, since this compound was identified as a modulator of PrP aggregation in this study.

Compound **7** exhibited a $[M-H]^-$ ion at m/z 593.1544, eluting at 24.4 minutes. This compound was tentatively identified as vicenin-2, a di-*C*-glycosyl flavonoid, based on the fragmentation data. The fragment ion at m/z 473.1094 involved the neutral loss of $C_4H_8O_4$ moiety ($^{0,2}X1$ 120 Da) from the $[M-H]^-$ ion, while the base peak at m/z 353.0674 resulted from a further loss of another $C_4H_8O_4$ unit ($^{0,2}X$, 120 Da), both from the cross-ring cleavage of the hexose units. The fragment at m/z 383.0779 was formed upon the loss of $C_4H_8O_4$ unit ($^{0,2}X$, 120 Da) and $C_3H_6O_3$ moiety ($^{0,3}X$, 90 Da) from the $[M-H]^-$ ion.[8,9]

Compound **9,** tentatively identified as isoorientin (luteolin-6-*C*-glucoside), a C-glucosyl flavone consisting of luteolin having a β-*D*-glucosyl residue at the 6-position, eluted at 26.5 minutes with $[M-H]^-$ ion at m/z 447.0933. Its fragment ions at m/z 357.0567 ($^{0,3}X$), m/z 327.0517 ($^{0,2}X$), and m/z 297.0388 ($^{0,1}X$) indicate the loss of $C_3H_6O_3$ (90 Da), $C_4H_4O_4$ (120 Da), and $C_5H_{10}O_5$ (150 Da) from the $[M-H]^-$ ion, respectively, consistent with cross-ring cleavage of the glucose moiety.[10]

Compound **10** exhibited $[M-H]^-$ ions at m/z 625.1415 and a retention time of 26.7 minutes, annotated as quercetin-3,4-*O*-di-β-hexose. Fragment ions at m/z 463.0880 and 301.0354 indicate the loss of one (162 Da) and two (324 Da) hexose residues, respectively.[11]

Compounds **11** and **12** are isomers and yielded $[M-H]^-$ ions at m/z 431.0980. These compounds eluted at 27.2 and 28 minutes, respectively, and were identified as apigenin flavone glycosides, specifically vitexin (compound **11**) and isovitexin (compound **12**). The product ion spectra of both compounds ions show fragments at m/z 311.0563 ($^{0,2}X$) and 341.0659 ($^{0,3}X$), indicating the loss of $C_4H_4O_4$ (120 Da) and $C_3H_6O_3$ (90 Da) from the $[M-H]^-$ ion, respectively. These losses are consistent with cross-ring cleavage of the glucose moiety in the flavonoid glycosyl

unit. Fragment ions at m/z 283.0616 correspond to the benzyl ion, which is characteristic of the fragmentation patterns of both vitexin and isovitexin. However, the product ion spectra differ in the relative abundance of the m/z 341.0656 ion, which is more intense in the isovitexin spectrum. Additionally, the product ion at m/z 353.0670 is observed exclusively in the isovitexin spectrum.[12,13] Therefore, compound **11** was tentatively identified as vitexin, while compound **12** was characterized as isovitexin.

Compound **13**, tentatively identified as rutin, exhibited $[M+H]^+$ ions at 611.1622 with a retention time of 29.1 minutes. In the positive ionization mode, the product ion at m/z 465.1042 was formed by the loss of one rhamnose from the $[M+H]^+$ ion. This fragment further dissociated to produce the ion at m/z 303.0515, corresponding to the loss of glucose, which resulted in the base peak. Fragment ions at m/z 129.0571, corresponding to the oxonium ion ($C_6H_9O_3^+$), are characteristic of rhamnose-containing compounds.[14]

Compound **14** exhibited $[M+H]^+$ ions at 507.1197 and a retention time of 30.4 minutes, tentatively identified as quercetin-3-*O*-hexose-6''-acetate. The base peak at m/z 303.0506 is produced by losing the hexose-6-acetate moiety (204 Da).

Compound **15**, tentatively identified as kaempferol-7-*O*-hexose, eluted at 30.8 minutes and exhibited $[M+H]^+$ ions at m/z 595.1672. The product ion at m/z 449.1081 results from the cleavage of one glycosidic bond, while the base peak at m/z 287.0557 is formed by the dissociation of both glycosidic bonds.[15]

Compound **16** was identified as isorhamnetin-3-*O*-hexose, with a retention time of 30.95 minutes, showing $[M+H]^+$ ions at m/z 625.1801. The product ions at m/z and 479.1197 are produced by the cleavage of the glycosidic bond, resulting in the loss of neutral fragments $C_6H_{10}O_4$ (146 Da). The dissociation of both glycosidic bonds results in the loss of neutral fragments $C_{12}H_{19}O_9$ (308 Da), producing ions at m/z 317.0665.

Compound **17** exhibited $[M-H]^-$ ions at 549.0897 and a retention time of 31.7 minutes, tentatively identified as quercetin-3-*O*-malonylhexose. The base peak at m/z 505.1003 is produced by losing CO_2, while the product ion at m/z 300.0287 is formed by fragmentation of the glycosidic bond, resulting in the loss of the malonyl hexose.

Compound **18**, tentatively identified as luteolin 7-(6''-malonylhexose), eluted at 33.5 minutes and exhibited $[M-H]^-$ ions at m/z 533.0941. The base peak at m/z 489.1044 is produced by the

loss of CO_2, while the cleavage of the glycosidic bond with the loss of the malonyl-hexose results in the formation of the ion product at m/z 285.0400.[16]

In total, 18 compounds were annotated, comprising one nucleoside, one amino acid, five phenolic acids, and eleven flavonoids. Given that *M. oleifera* is a well-studied plant known for its pharmacological activities, most of these compounds have already been reported in its leaves. However, compound 9, tentatively identified as isoorientin, had only been previously described in the seeds of *M. oleifera.*[17] Additionally, compound 18, characterized as luteolin 7-(6''-malonylhexose), appears to be reported here for the first time in the leaves of *M. oleifera*, based on our literature review. These findings expand the chemical profile of *M. oleifera* leaves and contribute to provide a deeper understanding of its bioactive potential.

References

(1) Schmid, R.; Heuckeroth, S.; Korf, A.; Smirnov, A.; Myers, O.; Dyrlund, T. S.; Bushuiev, R.; Murray, K. J.; Hoffmann, N.; Lu, M.; Sarvepalli, A.; Zhang, Z.; Fleischauer, M.; Dührkop, K.; Wesner, M.; Hoogstra, S. J.; Rudt, E.; Mokshyna, O.; Brungs, C.; Ponomarov, K.; Mutabdžija, L.; Damiani, T.; Pudney, C. J.; Earll, M.; Helmer, P. O.; Fallon, T. R.; Schulze, T.; Rivas-Ubach, A.; Bilbao, A.; Richter, H.; Nothias, L. F.; Wang, M.; Orešič, M.; Weng, J. K.; Böcker, S.; Jeibmann, A.; Hayen, H.; Karst, U.; Dorrestein, P. C.; Petras, D.; Du, X.; Pluskal, T. Integrative Analysis of Multimodal Mass Spectrometry Data in MZmine 3. *Nature Biotechnology*. Nature Research April 1, 2023, pp 447–449. https://doi.org/10.1038/s41587-023-01690-2.

(2) Wang, M.; Carver, J. J.; Phelan, V. V.; Sanchez, L. M.; Garg, N.; Peng, Y.; Nguyen, D. D.; Watrous, J.; Kapono, C. A.; Luzzatto-Knaan, T.; Porto, C.; Bouslimani, A.; Melnik, A. V.; Meehan, M. J.; Liu, W. T.; Crüsemann, M.; Boudreau, P. D.; Esquenazi, E.; Sandoval-Calderón, M.; Kersten, R. D.; Pace, L. A.; Quinn, R. A.; Duncan, K. R.; Hsu, C. C.; Floros, D. J.; Gavilan, R. G.; Kleigrewe, K.; Northen, T.; Dutton, R. J.; Parrot, D.; Carlson, E. E.; Aigle, B.; Michelsen, C. F.; Jelsbak, L.; Sohlenkamp, C.; Pevzner, P.; Edlund, A.; McLean, J.; Piel, J.; Murphy, B. T.; Gerwick, L.; Liaw, C. C.; Yang, Y. L.; Humpf, H. U.; Maansson, M.; Keyzers, R. A.; Sims, A. C.; Johnson, A. R.; Sidebottom, A. M.; Sedio, B. E.; Klitgaard, A.; Larson, C. B.; Boya, C. A. P.; Torres-Mendoza, D.; Gonzalez, D. J.; Silva, D. B.; Marques, L. M.; Demarque, D. P.; Pociute, E.; O'Neill, E. C.; Briand, E.; Helfrich, E. J. N.; Granatosky, E. A.; Glukhov, E.; Ryffel, F.; Houson, H.; Mohimani, H.; Kharbush, J. J.; Zeng, Y.; Vorholt, J. A.; Kurita, K. L.; Charusanti,

P.; McPhail, K. L.; Nielsen, K. F.; Vuong, L.; Elfeki, M.; Traxler, M. F.; Engene, N.; Koyama, N.; Vining, O. B.; Baric, R.; Silva, R. R.; Mascuch, S. J.; Tomasi, S.; Jenkins, S.; Macherla, V.; Hoffman, T.; Agarwal, V.; Williams, P. G.; Dai, J.; Neupane, R.; Gurr, J.; Rodríguez, A. M. C.; Lamsa, A.; Zhang, C.; Dorrestein, K.; Duggan, B. M.; Almaliti, J.; Allard, P. M.; Phapale, P.; Nothias, L. F.; Alexandrov, T.; Litaudon, M.; Wolfender, J. L.; Kyle, J. E.; Metz, T. O.; Peryea, T.; Nguyen, D. T.; VanLeer, D.; Shinn, P.; Jadhav, A.; Müller, R.; Waters, K. M.; Shi, W.; Liu, X.; Zhang, L.; Knight, R.; Jensen, P. R.; Palsson, B.; Pogliano, K.; Linington, R. G.; Gutiérrez, M.; Lopes, N. P.; Gerwick, W. H.; Moore, B. S.; Dorrestein, P. C.; Bandeira, N. Sharing and Community Curation of Mass Spectrometry Data with Global Natural Products Social Molecular Networking. *Nature Biotechnology*. Nature Publishing Group September 8, 2016, pp 828–837. https://doi.org/10.1038/nbt.3597.

(3) de Faria, R. A.; Oliveira, P. C. O.; de Carvalho, M. D. P.; Peixoto, B. S.; Severino, V. G. P.; Tinoco, L. W.; Rodrigues, S. V.; de Moraes, M. C. High-Resolution Inhibition Profiling and Ligand Fishing for Screening of Nucleoside Hydrolase Ligands in Moringa Oleifera Lamarck. *J. Pharm. Biomed. Anal.* **2022**, *211*, 114614. https://doi.org/10.1016/j.jpba.2022.114614.

(4) Zhang, P.; Chan, W.; Ang, I. L.; Wei, R.; Lam, M. M. T.; Lei, K. M. K.; Poon, T. C. W. Revisiting Fragmentation Reactions of Protonated α-Amino Acids by High-Resolution Electrospray Ionization Tandem Mass Spectrometry with Collision-Induced Dissociation. *Sci. Rep.* **2019**, *9* (1). https://doi.org/10.1038/s41598-019-42777-8.

(5) Pearson, J. L.; Lee, S.; Suresh, H.; Low, M.; Nang, M.; Singh, S.; Lamin, F.; Kazzem, M.; Sullivan, S.; Khoo, C. S. The Liquid Chromatographic Determination of Chlorogenic and Caffeic Acids in Xu Duan (Dipsacus Asperoides) Raw Herb . *ISRN Anal. Chem.* **2014**, *2014*, 1–6. https://doi.org/10.1155/2014/968314.

(6) Clifford, M. N.; Johnston, K. L.; Knight, S.; Kuhnert, N. Hierarchical Scheme for LC-MSn Identification of Chlorogenic Acids. *J. Agric. Food Chem.* **2003**, *51* (10), 2900–2911. https://doi.org/10.1021/jf026187q.

(7) Yang, J.; Yao, L.; Gong, K.; Li, K.; Sun, L.; Cai, W. Identification and Quantification of Chlorogenic Acids from the Root Bark of Acanthopanax Gracilistylus by UHPLC-Q-Exactive Orbitrap Mass Spectrometry. *ACS Omega* **2022**, *7* (29), 25675–25685. https://doi.org/10.1021/acsomega.2c02899.

(8) Hong, Y.; Liao, X.; Chen, Z. Determination of Bioactive Components in the Fruits of

Cercis Chinensis Bunge by HPLC-MS/MS and Quality Evaluation by Principal Components and Hierarchical Cluster Analyses. *J. Pharm. Anal.* **2021**, *11* (4), 465–471. https://doi.org/10.1016/j.jpha.2020.07.010.

(9) Silva, D. B.; Turatti, I. C. C.; Gouveia, D. R.; Ernst, M.; Teixeira, S. P.; Lopes, N. P. Mass Spectrometry of Flavonoid Vicenin-2, Based Sunlight Barriers in Lychnophora Species. *Sci. Rep.* **2014**, *4*. https://doi.org/10.1038/srep04309.

(10) Shao, S. Y.; Ting, Y.; Wang, J.; Sun, J.; Guo, X. F. Characterization and Identification of the Major Flavonoids in Phyllostachys Edulis Leaf Extract by UPLC-QTOF-MS/MS. *Acta Chromatogr.* **2020**, *32* (4), 228–237. https://doi.org/10.1556/1326.2019.00688.

(11) Francescato, L. N.; Debenedetti, S. L.; Schwanz, T. G.; Bassani, V. L.; Henriques, A. T. Identification of Phenolic Compounds in Equisetum Giganteum by LC-ESI-MS/MS and a New Approach to Total Flavonoid Quantification. *Talanta* **2013**, *105*, 192–203. https://doi.org/10.1016/j.talanta.2012.11.072.

(12) Krasteva, I.; Nikolov, S. *FLAVONOIDS IN Astragalus Corniculatus*; 2008; Vol. 31.

(13) Waridel, P.; Wolfender, J.-L.; Ndjoko, K.; Hobby, K. R.; Major, H. J.; Hostettmann, K. Evaluation of Quadrupole Time-of-Flight Tandem Mass Spectrometry and Ion-Trap Multiple-Stage Mass Spectrometry for the Differentiation of C-Glycosidic Flavonoid Isomers. *J. Chromatogr. A* **2001**, *926* (1), 29–41. https://doi.org/https://doi.org/10.1016/S0021-9673(01)00806-8.

(14) Wang, J.; Ren, X.; Wen, C.; Xu, Y.; Chen, Y. Separation and Characterization of Unknown Impurities in Rutin Tablets Using Trap-Free Two-Dimensional Liquid Chromatography Coupled with Ion Trap/Time-of-Flight Mass Spectrometry. *Rapid Commun. Mass Spectrom.* **2020**, *34* (10), e8739. https://doi.org/https://doi.org/10.1002/rcm.8739.

(15) Ma, Y.-L.; Vedernikova, I.; Van den Heuvel, H.; Claeys, M. Internal Glucose Residue Loss in Protonated O-Diglycosyl Flavonoids upon Low-Energy Collision-Induced Dissociation. *J. Am. Soc. Mass Spectrom.* **2000**, *11* (2), 136–144. https://doi.org/10.1016/S1044-0305(99)00133-6.

(16) Li, Z. H.; Guo, H.; Xu, W. Bin; Ge, J.; Li, X.; Alimu, M.; He, D. J. Rapid Identification of Flavonoid Constituents Directly from PTP1B Inhibitive Extract of Raspberry (Rubus Idaeus L.) Leaves by HPLC-ESI-QTOF-MS-MS. *J. Chromatogr. Sci.* **2016**, *54* (5), 805–810. https://doi.org/10.1093/chromsci/bmw016.

(17) Premi, M.; Sharma, H. K. Effect of Extraction Conditions on the Bioactive Compounds

from Moringa Oleifera (PKM 1) Seeds and Their Identification Using LC–MS. *J. Food Meas. Charact.* **2017**, *11* (1), 213–225. https://doi.org/10.1007/s11694-016-9388-y.
